# Nutrition Assessment of Under-Five Children in Sudan: Tracking the Achievement of the Global Nutrition Targets

**DOI:** 10.3390/children8050363

**Published:** 2021-05-01

**Authors:** Mohamed Abu-Manga, Ayoub Al-Jawaldeh, Abdul Baseer Qureshi, Amira M. Elmunier Ali, Damiano Pizzol, Fekri Dureab

**Affiliations:** 1 Federal Ministry of Health, National Nutrition Program, Khartoum 11111, Sudan; mohabumanga80@gmail.com; 2 Regional Office for the Eastern Mediterranean (EMRO), World Health Organization (WHO), Cairo 7608, Egypt; aljawaldeha@who.int; 3 World Health Organization (WHO), Khartoum 11111, Sudan; qureshiab@who.int (A.B.Q.); alia@who.int (A.M.E.A.); 4 Italian Agency for Development Cooperation, Khartoum 11111, Sudan; damianopizzol8@gmail.com; 5 Heidelberg Institute of Global Health, Hospital University Heidelberg, 69120 Heidelberg, Germany; 6 Institute for Research in International Assistance, Akkon-Hochschule für Humanwissenschaften, 12099 Berlin, Germany

**Keywords:** malnutrition, global nutrition targets, Sudan, S3M survey

## Abstract

Background: Malnutrition places a heavy burden on the health, well-being, and sustainable development of populations in Sudan, especially a country affected by conflict, which continues to experience high levels of food insecurity, undernutrition, and micronutrient deficiencies; 3.3 million are acutely malnourished, with 522,000 children suffering from severe acute malnutrition and approximately 2.2 million children requiring treatment for moderate acute malnutrition. This study aims to describe the nutritional status of children under five years old and identify the progress toward the achievement of the Global Nutrition Targets. Methods: This is a secondary data analysis of a quantitative survey, using the second-round of the Simple Spatial Survey Method (S3M II) in Sudan in the period 2018–2019. The analysis used an area-based sampling methodology in all 18 Sudanese states. Data from the WHO Tracking Tools of the Global Nutrition Targets was used to reflect the progress in achieving the targets in Sudan. Results: Global stunting prevalence was at 36.35 percent including moderate stunting prevalence and severe stunting prevalence (21.25 percent and 15.06 percent respectively). Global wasting prevalence was 13.6 percent including moderate wasting prevalence and severe wasting prevalence (10.8 percent and 2.7 percent respectively). Sudan has made great progress in achieving the target of increasing exclusive breastfeeding. However, despite the welcome commitments by the Government and all stakeholders, Sudan is still struggling to implement strategies, policies, and regulatory measures to address malnutrition and achieve the Global Nutrition Targets in 2025 and the Sustainable Developmental Goals in 2030. Therefore, more than ever, there is a need for comprehensive, multi-sectoral action to address malnutrition in all its forms.

## 1. Introduction

Malnutrition is the main cause of death and diseases in the world and undernutrition is responsible for about 45% of all deaths of under five children. These deaths mainly occur in low- and middle-income countries where child obesity is increasing at the same time [1,2]. Globally, about 47 million (6.9%) under five children are acutely malnourished (wasted) and 14.3 million of them are severely wasted. Approximately, 144 million (21.3%) are stunted, while 38.3 million (5.6%) are overweight or obese [3,4].

Nutrition is a prominent topic on global and national agendas. The Global Nutrition Targets that should be achieved by 2025 include six targets that aim (1) to reduce the number of stunted under five children by 40%, (2) to reduce and maintain wasting in children to less than 5%, (3) to reduce anemia in women of reproductive age by 50%, (4) to decrease low birth weight by 30%, and (5) to increase exclusive breastfeeding in the first 6 months to, at least, 50% [5,6].

Sudan is an Arab country located in northeast Africa with a long history of protracted social conflicts that have had a clear impact on basic infrastructures, social services, and the general health and nutritional status of the population, in particular children [7,8]. The United Nations estimated 9.3 million people in Sudan needed some kind of humanitarian assistance in 2020. Health care is the most required service by the vulnerable population in Sudan with 8.6 million people lacking adequate healthcare. About 3.3 million people are acutely malnourished, with 522,000 children suffering from severe acute malnutrition (SAM) and approximately 2.2 million children requiring treatment for moderate acute malnutrition (MAM). About 7.6 million people lack access to safe water, sanitation, and hygiene services [9].

Moreover, Sudan is actually facing a food security emergency due to the macroeconomic crisis that increased the national inflation rate to 144 percent in 2020 and devastated the purchasing power of the Sudanese. Furthermore, the COVID-19 outbreak, flooding, civil conflicts, and the Desert Locust are likely to contribute in increasing the price of the staple foods in the country [10,11]. An average price of a food basket costs at least 75 percent of household income [12]. According to the Integrated Food Security Phase Classification (IPC), 16% of the population (7.1 million) is classified in a crisis phase and above (IPC phase 3 and 4) [13].

Considering that information on the nutritional status of children in Sudan is very scarce, this article gives an overview of the most important nutritional indicators of children in all 18 States of Sudan. It aims to describe the prevalence of malnutrition of under-fives children in relation to anthropometric and clinical standards and to describe the infant and child feeding practices, in addition, to investigating the progress of Sudan toward the achievement of the Global Nutrition Targets.

## 2. Materials and Methods

This manuscript is a secondary data analysis of a quantitative descriptive study, based on the data from the second-round of the Simple Spatial Survey Method (S3M II) in Sudan in the period 2018–2019. In this manuscript, we used two types of data resources. First, the data from the S3M II report to describe the nutrition indicators in children under five years-old [14]. Second, the data from the Tracking Tools of the Global Nutrition Targets was used (stunting, wasting, overweight, and exclusive breastfeeding) to reflect the progress of achieving the targets in Sudan [15]. We compared the findings of the S3M II survey with the baseline data of 2010 in the dashboard of the Tracking Tools. We compared the findings of S3M II with the findings of S3M I in the discussion to have an overview on the changes occurring on the nutritional status of children in the period between 2013 and 2018. Both surveys adapted the same methodology. However, the overall sample size increased in S3M II compared to S3M I, which was mainly due to improved access to the villages.

### 2.1. Methods of S3M Surveys

The survey S3M II used an area-based sampling methodology in all 18 Sudanese states. It gathered data on 230 critical indicators regarding the following broad topics: health; nutrition; water, sanitation, and hygiene (WASH) as well as child and social protection. The nutritional status of children aged 6–59 months in S3M II was assessed via anthropometric measurements of weight, height, and mid-upper-arm circumference (MUAC) using standard measurement tools; Seca electronic scale bat.mains.solar for weighing, portable L-hgt mea.syst/SET-2 for height, and Children’s Mid Upper Arm Circumference measuring tape with cut-off point at 11.5 cmt. Using weight and height measurements, the corresponding nutritional indices of weight-for-age Z-score (WAZ), height-for-age Z-score (HAZ), and weight-for-height Z-score (WHZ) were calculated using the WHO Child Growth Standards (WGS) as reference standard [16], to determine the child’s underweight status, stunting status, and wasting status, respectively. Table 1 shows the standard thresholds for wasting, stunting, and overweight. The Z-scores were calculated using the Z-scorer package in R software. MUAC, on the other hand, was used as-is without standardization and assessed based on accepted cut-offs for wasting status [17]. Finally, nutritional oedema was assessed clinically using the bilateral pitting oedema test. Following this the prevalence of various forms of undernutrition in children 6–59 months are given.

The survey S3M I was carried out in all 18 states of Sudan using the same methodology of area-based sampling. A total of 45,094 households and 71,625 children below 5 years of age were surveyed. Data collection took place during June/July 2013 for 14 states and in November 2013 for the remaining 4 states (Khartoum, Red Sea, South and West Kordofan) in Sudan. A total of 59 indicators was measured covering child and maternal health and nutrition as well as WASH services [18].

### 2.2. Tracking Tools of the Global Nutrition Targets

The WHO, in collaboration with United Nations Children’s Fund (UNICEF) and the European Commission (EC), developed the Tracking Tool to help countries set their national targets and monitor progress. This tool allows users to explore scenarios taking into account different rates of progress for the six global targets and the time remaining until 2025. The baseline data for Sudan was taken from the household health survey, second round 2010 [15].

### 2.3. The Sample

The S3M survey was designed to be spatially representative of the whole country, including its smaller administrative units up to the locality level, except for a few inaccessible areas. The selection of sample units was performed based on random sample selection using sampling software designed to undertake S3M variable density sampling. An even distribution of primary sampling units (PSUs) (i.e., villages/city blocks) was selected from across the country. About 31, 32, 33 PSUs (i.e., villages/city blocks) were selected based on their proximity to centroids of a hexagonal grid laid over the entire country. Across Sudan, a total of 93,882 households and 145,002 children below 5 years of age were surveyed. To select PSUs, the map-segment-sample approach was used for within-community sampling of PSUs. In this approach, PSUs organized as ribbons of dwellings were sampled systematically, while those organized as clusters of dwellings were sampled using a random walk strategy [21,22].

### 2.4. Data Collection

The S3M II survey collected data in two phases. Data from phase one states (North Darfur, East Darfur, West Kordofan, River Nile, Sennar, South Darfur, North Kordofan, Khartoum and Northern states) in Sudan was collected in October 2018. Data from phase two states (White Nile, Kassala, Blue Nile, Central Darfur and West Darfur, Red Sea, South Kordofan and Gedaref states) in Sudan was collected from November 2018 to January 2019.

### 2.5. Definitions of Nutrition Indicators

1—Wasting is acute malnutrition reflected by weight-for-height Z-score (WHZ) or MUAC. Global Acute Malnutrition GAM includes Severe Acute malnutrition (SAM) and Moderate Acute Malnutrition (MAM).

➢ Wasting global (WHZ) is the percentage of children 6–59 months < −2 Z-scores weight for height and/or oedema.
  - Wasting severe (WHZ) is the percentage of children 6–59 months < −3 Z-scores weight for height and/or oedema.
  - Wasting Moderate (WHZ) is the percentage of children 6–59 months between < −2 Z-scores and −3 z-scores weight for height.
➢ Wasting global (MUAC) is the percentage of children 6–59 months with an MUAC < 125 mm and or oedema.
  - Wasting severe (MUAC) is the percentage of children 6–59 months with a MUAC < 115.
  - Wasting Moderate (MUAC) is the percentage of children 6–59 months with a MUAC between <1 25 mm and > 115 mm.

2—Stunting is chronic malnutrition reflected by height-for-age Z-score (HAZ).

➢ Stunting global (HAZ) is the percentage of children 6–59 months < −2 Z-scores height for age.
  - Stunting severe (HAZ) is the percentage of children 6–59 months < −3 Z-scores height for age.
  - Stunting Moderate (HAZ) is the percentage of children 6–59 months between < −2 Z-scores and −3 z-scores height for age.

3—Underweight is the percentage of children who have low weight for age Z-score (WAZ),

➢ Underweight global (WAZ) is the percentage of children 6–59 months < −2 Z-scores weight for age.
  - Underweight severe (WAZ) is the percentage of children 6–59 months < −3 Z-scores weight for age.
  - Underweight Moderate (WAZ) is the percentage of children 6–59 months between < −2 Z-scores and −3 z-scores weight for age.

4—Overweight/Obesity is the percentage of children 6–59 months >2 Z-scores weight for height/age.
5—Nutritional oedema was assessed clinically using the bilateral pitting oedema test.
6—Exclusive breastfeeding is the percentage of children 0–6 months taking breast milk only.
7—Continuing Breast-feeding is the percentage of children 6–24 months currently breastfeeding.
8—Meal frequency is the percentage of children receiving meals the appropriate number of times for their age during the 24 h before the survey.
9—Dietary diversity is the percentage of children consuming the appropriate number of food groups for their age during the 24 h before the survey.

## 3. Results

The mean HAZ of children 6–59 months old in Sudan was −1.49 (95 percent Confidence Interval (CI): −1.71 to 1.28) Z-score. Among the states, the mean HAZ was highest in Khartoum state at −1.07 (95 percent CI: −1.3 to 0.85) Z-score while Red Sea state had the lowest mean HAZ at −1.90 (95 percent CI: −2.18 to 1.62) see Table 2. However, among the localities, four have a significantly lower mean HAZ: Refi Hamashkureib (−2.74) and Rural Telkok (−2.51) in Kassala state; El Ganab Elawlaib (−2.61) in Red Sea state; and Sharg El Jabal (−2.59) in South Darfur.

Global stunting prevalence (Overall national rate) was at 36.35 percent. Moderate stunting prevalence was at 21.25 percent. Severe stunting prevalence was at 15.06 percent. Among the states, Red Sea state had the highest prevalence of global (48.4 percent) and severe stunting (24.8 percent), followed by Kassala (47.7 percent). While North Darfur had global prevalence of stunting of 45.8 percent and the highest prevalence of moderate stunting (25 percent). South Darfur state had the lowest prevalence of global (23.7 percent) and severe stunting (6.8 percent) and Khartoum had the lowest prevalence of moderate stunting (16.6 percent) see Table 2. Of all the localities, 49.5 percent had global stunting prevalence greater than 40 percent, particularly some localities in Kassala state and South Darfur state face an extremely high prevalence of stunting.

The mean weight-for-age Z-score (WAZ) of children 6–59 months old in Sudan was −1.41 (95 percent CI: −1.59 to 1.24) Z-score. Among the states, the mean WAZ was highest in Khartoum state at −1.19 (95 percent CI: −1.37 to 1.01) Z-score, while Red Sea state had the lowest mean WAZ at −2.02 (95 percent CI: −2.22 to 1.81) see Table 3. Among the localities, Shia-ria locality in East Darfur state and Sinkat, Haya and Tokar locality in Red Sea state had the lowest mean WAZ of all localities.

Global underweight prevalence was at 29.16 percent. Moderate underweight prevalence was 21.14 percent. Severe underweight prevalence was at 7.93 percent. Among the states, Red Sea state had the highest prevalence of global (49.77 percent) and severe underweight (23.33 percent). While North Darfur had the highest prevalence of moderate underweight (29.54 percent). On the other hand, Blue Nile state had the lowest prevalence of global (21.30 percent) and moderate underweight (16.93 percent), and South Darfur had the lowest prevalence of severe underweight (3.97 percent) see Table 3. Of all the localities, 89.4 percent had global underweight prevalence greater than 20 percent.

Table 4 shows the mean weight-for-height Z-score (WHZ) of children 6–59 months old in Sudan was –0.76 (95 percent CI: –0.96 to –0.57). Among the states, the mean WHZ was highest in Blue Nile state at –0.44 (95 percent CI: –0.61 to –0.27) Z-score, while Red Sea state had the lowest mean WHZ at –1.12 (95 percent CI: –1.4 to –0.83). Among the localities, El Rahad locality in Al-Gadarif state and Foro Baranga locality in West Darfur state had the highest mean WHZ at 0.1. Shia-ria locality in East Darfur state and Haya locality in Red Sea state had a significantly lower mean WHZ than all other localities. The mean MUAC of children 6–59 months old in Sudan was 141.44 (139.66143.21) mm. Among the states, the mean MUAC was highest in Khartoum state at 146.16 (95 percent CI: 144.24 to 148.08) mm, while Red Sea state had the lowest mean MUAC at 132.2 (95 percent CI: 129.99 to 134.42) mm (see Table 5).

Global wasting by WHZ prevalence was 13.6 percent. Moderate wasting by WHZ prevalence was 10.8 percent. Severe wasting by WHZ prevalence was 2.7 percent. These values were higher than the wasting prevalence by MUAC which showed wasting of 9.5 percent, moderate wasting of 7.6 percent and severe wasting of 1.8 percent see Table 5. There was no single state in Sudan with wasting prevalence less than the WHO medium wasting threshold of 5 percent. Among the states, Red Sea state had the highest prevalence of global and severe wasting by WHZ and MUAC, while North Darfur had the highest prevalence of moderate wasting by WHZ. On the other hand, Blue Nile state had the lowest prevalence of global, moderate, and severe wasting by WHZ. Of all the localities, 34 percent had global wasting prevalence greater than the 15 per cent threshold considered to be very high.

Nutritional oedema prevalence was at 0.18 percent (95 per cent CI: 0.01 to 0.86 per cent) across Sudan. West Darfur and North Darfur states had the highest prevalence of nutritional oedema (0.69 percent, 0.66 percent respectively), significantly higher than all other states.

Child overweight prevalence was 2.14 percent (95 percent CI: 0.01 to 4.42 percent) across Sudan. Child obesity prevalence was 0.85 percent (95 percent CI: 0.01 to 2.66 percent). Among the states, Al-Gadarif state had the highest prevalence of overweight (4.33 percent) and obese (1.74 percent) children.

The national exclusive breast-feeding prevalence (up to six months of age) was 62.31 percent (95 percent CI: 44.0 to 80.6 percent), with 73.29 percent (95 percent CI: 65.1 to 81.5 percent) age-appropriate continued breast-feeding up to 2 years of age or beyond, 63.24 percent (95 percent CI: 52.5 to 74.0 percent) age-appropriate meal frequency and 25.40 percent (95 percent CI: 17.0 to 33.8 percent) age-appropriate dietary diversity. Across the states, exclusive breast-feeding was highest in West Darfur state (81.01 percent) and lowest in River Nile state (49.36 percent) Age-appropriate continuing breast-feeding was highest in Kassala state (82.37 percent) and lowest in South and Central Darfur state (67.24 percent and 67.94 percent respectively). Age-appropriate dietary diversity was highest in North Kordofan state (34.47 percent) and lowest in East Darfur state (7.39 percent). Age-appropriate meal frequency was highest in Northern state (84.03 percent) and lowest in East Darfur state (47.22 percent).

**Figure 1:**
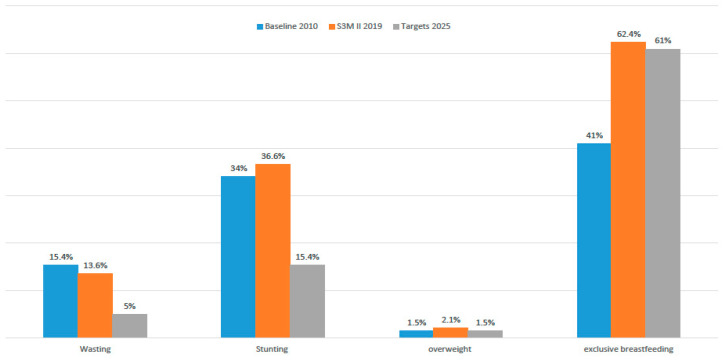
Tracking the Global Nutrition Targets based on the S3M II results.

Figure 1 reflects the result from S3M II focusing on four nutritional indictors; wasting, stunting, overweight, and exclusive breastfeeding, comparing to the baseline indicators of 2010 and the targets to be achieved in Sudan by 2025 from the WHO tracking tool of the Nutrition Global Targets. The results show that exclusive breastfeeding is the only indicator that has already been achieved (62.36%). Wasting is reduced slightly from 15.4% in 2010 to 13.6% in 2019, while stunting and overweight indictors are increased slightly compared to baseline values.

## 4. Discussion

In this study, we presented the prevalence of under-5 children undernutrition in Sudan based on the Simple Spatial Survey (S3M II). Knowing the prevalence rates of stunting, underweight, and wasting, in children under 5-years-old is essential for determining the overall nutrition status of the children and tracking the progress of Sudan toward the Global Nutrition Targets with particular focus on the children under five-years-old. The findings provide information for health partners and policymakers to ensure evidence-based, equity-focused programming and distribution of resources according to the priority areas and needs. All indicators of malnutrition in Sudan were noticeably high, the prevalence estimates for children under 5 years of age were for stunting 36.35%, for underweight 29.16%, and for wasting 11.36%.

The global stunting rate of children in Sudan of 36.4% in S3M II, indicates that there has been a slight improvement in general compared to the stunting rate of 38.2% in 2018, according to the report of the Multiple Indicator Cluster Survey 2014 and the Eastern Mediterranean Regional Office (EMRO) of the World Health Organization [23,24], however, it still higher than the baseline value in 2010. All states in Sudan had a stunting rate above the WHO very high-stunting threshold of 30 percent, except two states with a high-stunting threshold between 20–30 percent: Khartoum (25.2 percent) and South Darfur (23.7 percent). Comparing the results of S3M-2013 with S3M II showed that the stunting prevalence increased significantly in four places: North Darfur (from 35.2 percent to 45.8 percent); West Darfur (from 34.8 percent to 41.5 percent); River Nile (from 28.4 percent to 39 percent); and in Khartoum state (from 20.2 percent to 25.2 percent). In contrast, the stunting prevalence significantly decreased in three states: Kassala (from 54.6 percent to 47.8 per cent); Al-Gadarif (from 52 percent to 45.2 per cent); and Blue Nile (from 49.8 percent to 37.2 percent) [21]. This survey reflected that 162 of the total 185 localities (88 percent) had a stunting rate above 20 percent. The stunting rate is much higher in children living in rural areas, for instance, a study was conducted in rural areas of the River Nile state in 2018 which showed that the prevalence of stunting among children aged 0–60 months was 42.5% [25]. All forms of undernutrition are prevalent in rural areas compared to urban areas in Sudan most likely due to the limited family income and education of the parents, in addition to the high prevalence of infectious diseases among children and lack of health services [26].

The underweight prevalence increased significantly in four states compared to the S3M results in 2013: Northern (from 14.9 percent to 26.8 percent); River Nile (from 23 percent to 33.2 percent); Khartoum (from 15.6 percent to 23.5 per cent); and West Darfur (from 22.3 percent to 26.7 percent). On the other hand, the underweight prevalence significantly decreased in the states of Blue Nile (from 37.7 percent to 21.3 percent) and Kassala (from 49.1 percent to 32.7 percent) [24].

The survey showed that levels of wasting (low weight for height WHZ and MUAC) were relatively higher and more pronounced in those states where data was collected during phase one of the data collection (North Darfur, East Darfur, West Kordofan, River Nile, Sennar, South Darfur, North Kordofan, Khartoum, and Northern states), than those in phase two (White Nile, Kassala, Blue Nile, Central Darfur and West Darfur, Red Sea, South Kordofan, and Gedaref states). This is likely due to the seasonal gap of time between the data collection periods, as the first phase overlapped more with the lean season in Sudan. The results of this survey showed that global wasting by WHZ increased significantly in two states comparing to S3M I in 2013: Northern (from 7.3 percent to 16.5 percent); and Khartoum (from 8.2 percent to 14.6 percent). In contrast, the global wasting prevalence by WHZ significantly decreased in three states: Blue Nile (from 18.5 percent to 7.0 percent); Kassala (from 15.2 percent to 9.4 percent); and North Darfur (from 28.3 percent to 19.1 percent) [24]. Similarly, the variation between the prevalence in S3M 2013 and S3M 2018 could be due to the seasonal differences of the data collection period, however, poverty and the deterioration of the Sudanese economy cannot be ignored.

Malnutrition is a risk threatening vulnerable people due to multiple factors related to poverty and food security in the country in addition to the spread of diseases and several social factors that negatively affect the nutritional status of society [27,28]. The current crises of COVID-19 may deepen the problem of malnutrition in Sudan due to sharp drops in family incomes, difficulty with food availability and affordability, and disruptions of health, nutrition, and social protection services [29].

With the persistence of the usual problems of under nutrition in Sudan, there has been a gradual increase in overweight among children; findings showed that overweight increased from 1.5% in 2010 to 2.14% in 2019. The double burden of malnutrition is increasing in low and middle-income countries that are associated with advanced technology and transportation in reducing physical activities and increasing access to cheap fast food and beverages [30,31].

Ending malnutrition is important for society development. Achieving the Global Nutrition Targets is the ultimate goal to reach the United Nations’ Sustainable Development Goals (SDG), particularly SDG-2, zero hunger, which promises to end malnutrition in all its forms by the year 2030 [31]. Sudan has made great progress in achieving the target of increasing exclusive breastfeeding. However, despite these welcome commitments by the Government and all stakeholders, Sudan is still struggling to implement strategies, policies, and regulatory measures to address malnutrition to achieve the Global Nutrition Targets in 2025 and SDG goals in 2030. Shekar and her colleagues argued that achieving some of these targets globally is ambitious, particularly with regard to stunting and anemia in women [32].

There are limitations that may face any field survey in developing countries, especially those with unstable security, which may affect the collection and processing of data in some cases. In addition to the problem of recalling information among respondents, for example, there may be a recall bias when asking about exclusive breastfeeding. To avoid the recall bias in S3M, the questions focused on mothers of children under six months enquiring about exclusive breastfeeding over the last 24 h. Conducting the survey in different seasons may have caused a variation in the survey results between the target areas. Due to the unavailability of Child Growth Standards that are specific to Sudanese, the WHO Child Growth Standards was used as a reference. In addition to that there was no data available to elaborate more on the characteristics of the target population.

## 5. Conclusions

All indicators of malnutrition are high for Sudan among children under 5 years old. Global stunting prevalence was at 36.35 percent. Moderate stunting prevalence was at 21.25 percent. Severe stunting prevalence was at 15.06 percent. Global wasting by WHZ prevalence was 13.6 percent. Moderate wasting by WHZ prevalence was 10.8 percent. Severe wasting by WHZ prevalence was 2.7 percent. Progress on ending malnutrition in all its forms depends on action beyond the health sector. Considering this critical and urgent situation, it is mandatory that all stakeholders address child malnutrition as the central objective in the development agenda. In particular, national development frameworks should put in place a comprehensive multi-sectoral nutrition policy, a strategy and plan of action, with strong political commitment and allocation of adequate resources for its implementation across all line ministries.

Generally, nutrition services should be integrated with other essential services, for example, adapting cost effective interventions focusing on the critical 1000 day window of early childhood, requires access to safe water and sanitation. Growth monitoring programs and routine nutritional screening activities should be strengthened through community-based mechanisms with more focus on the eastern states. It is also important for the Government ensures continued availability of food and adequate medical prevention and treatment services in the context of COVID-19 considering that malnourished children are immunocompromised and high standards of infection control interventions are always recommended. Setting up triage protocols for COVID19 at all points of nutrition services and scaling up treatment at community level are urgently required.

## Figures and Tables

**Table 1:** Prevalence thresholds for public health significance for wasting, stunting, and overweight in children.

Labels	Wasting WHZ%	Overweight WAZ%	Stunting HAZ%
Very Low	<2.5	<2.5	<2.5
Low	2.5 < 5	2.5 < 5	2.5 < 5
Medium	5 < 10	5 < 10	5 < 10
High	10 < 15	10 < 15	10 < 15
Very High	>15	>15	>30
WHZ: weight-for-height Z-score, WAZ: weight-for-age Z-score, HAZ: Height-for-Age Z-score			

Source 1: New prevalence thresholds for stunting, wasting, and overweight in children’ [19]. Source 2: Prevalence thresholds for wasting, overweight, and stunting in children under 5 years [20].

**Table 2:** Distribution of stunting prevalence and mean by state.

State	Mean HAZ			HAZ < −2%			HAZ −3 to −2%			HAZ < −3%		
	Estimation	95% LCL	95% UCL	Estimation	95% LCL	95% UCL	Estimation	95% LCL	95% UCL	Estimation	95% LCL	95% UCL
Al Gedaref	−1.76	−1.98	−1.54	45.16	39.03	51.28	23.14	14.73	31.54	21.95	16.37	27.54
Al-Gezira	−1.46	−1.65	−1.26	35.04	29.56	40.52	22.51	15.53	29.49	12.42	7.66	17.19
Blue Nile	−1.50	−1.72	−1.29	37.23	31.26	43.20	21.13	13.46	28.79	16.08	10.90	21.27
Central Darfur	−1.65	−1.94	−1.36	41.95	32.56	51.34	20.96	9.20	32.71	20.93	12.62	29.24
East Darfur	−1.54	−1.79	−1.28	39.55	33.92	45.19	21.00	13.03	28.98	18.57	13.06	24.07
Kassala	−1.89	−2.06	−1.71	47.72	41.41	54.03	24.95	16.48	33.42	22.81	17.62	28.01
Khartoum	−1.07	−1.30	−0.85	25.19	18.03	32.34	16.59	8.35	24.82	8.52	4.42	12.61
North Darfur	−1.83	−2.07	−1.59	45.80	37.41	54.19	25.07	14.21	35.94	20.66	14.14	27.18
North Kordofan	−1.67	−1.83	−1.50	40.11	35.28	44.94	24.41	17.78	31.03	15.77	11.43	20.12
Northern	−1.20	−1.43	−0.96	30.17	22.77	37.56	19.40	10.89	27.92	10.86	5.56	16.15
Red Sea	−1.90	−2.18	−1.62	48.38	40.11	56.65	23.58	12.04	35.11	24.83	17.04	32.61
River Nile	−1.56	−1.76	−1.36	39.02	31.64	46.39	22.25	14.06	30.44	16.61	11.05	22.17
Sennar	−1.48	−1.69	−1.28	35.13	29.26	41.00	21.57	13.88	29.26	13.63	9.01	18.26
South Darfur	−1.16	−1.35	−0.96	23.68	16.17	31.19	16.81	8.19	25.42	6.78	2.56	10.99
South Kordofan	−1.67	−1.90	−1.44	40.77	31.75	49.79	22.80	12.21	33.39	17.81	11.28	24.35
West Darfur	−1.57	−1.78	−1.36	41.45	35.95	46.95	21.86	14.31	29.41	19.62	14.79	24.45
West Kordofan	−1.54	−1.73	−1.36	36.83	31.20	42.46	21.88	15.11	28.65	15.02	10.89	19.16
White Nile	−1.60	−1.81	−1.39	39.95	33.50	46.40	22.68	15.30	30.06	17.29	12.51	22.08
Sudan	−1.49	−1.71	−1.28	36.35	29.56	43.14	21.25	12.81	29.70	15.06	9.87	20.25

HAZ: Height-for-Age Z-score, LCL and UCL: Lower and Upper Confidence Interval Limits, (*n* = 145,002 children).

**Table 3:** Distribution of underweight prevalence and mean by state.

State	Mean WAZ			WAZ < −2%			WAZ −3 to −2%			WAZ < −3%		
	Estimation	95% LCL	95% UCL	Estimation	95% LCL	95% UCL	Estimation	95% LCL	95% UCL	Estimation	95% LCL	95% UCL
Al Gedaref	−1.42	−1.60	−1.24	29.98	23.87	36.08	21.64	13.98	29.30	8.26	3.81	12.72
Al-Gezira	−1.27	−1.42	−1.12	23.68	16.57	30.79	18.64	10.88	26.40	4.88	1.63	8.12
Blue Nile	−1.19	−1.33	−1.04	21.30	15.67	26.94	16.93	10.56	23.30	4.34	1.59	7.10
Central Darfur	−1.56	−1.80	−1.32	33.64	26.40	40.89	22.57	12.62	32.52	10.84	4.85	16.82
East Darfur	−1.60	−1.80	−1.39	35.97	29.47	42.47	23.73	15.51	31.96	12.10	7.03	17.17
Kassala	−1.51	−1.67	−1.35	32.71	25.82	39.60	23.80	15.74	31.86	8.86	4.44	13.27
Khartoum	−1.19	−1.37	−1.01	23.48	16.59	30.37	17.70	10.07	25.33	5.75	2.00	9.50
North Darfur	−1.85	−2.05	−1.64	44.01	33.47	54.56	29.54	17.30	41.79	14.44	7.25	21.64
North Kordofan	−1.55	−1.68	−1.43	32.55	27.30	37.81	24.71	18.55	30.86	7.84	4.84	10.85
Northern	−1.36	−1.54	−1.17	26.82	18.88	34.76	20.73	12.12	29.34	5.73	2.10	9.36
Red Sea	−2.02	−2.22	−1.81	49.77	40.16	59.38	26.19	13.37	39.01	23.33	14.31	32.35
River Nile	−1.54	−1.76	−1.33	33.20	25.91	40.48	22.89	13.94	31.85	10.19	5.06	15.31
Sennar	−1.32	−1.48	−1.17	25.78	19.33	32.23	19.92	12.98	26.86	5.67	2.50	8.83
South Darfur	−1.29	−1.45	−1.13	22.27	16.14	28.39	18.22	11.38	25.06	3.97	0.71	7.22
South Kordofan	−1.32	−1.50	−1.14	26.07	19.12	32.93	20.15	12.25	28.05	5.88	2.41	9.35
West Darfur	−1.34	−1.52	−1.16	26.71	19.72	33.70	20.12	12.39	27.85	6.32	2.86	9.79
West Kordofan	−1.47	−1.62	−1.31	30.74	24.46	37.02	22.50	14.73	30.26	8.32	3.98	12.67
White Nile	−1.31	−1.48	−1.15	26.49	20.28	32.70	19.89	12.50	27.28	6.46	2.93	10.00
Sudan	−1.41	−1.59	−1.24	29.16	22.05	36.27	21.14	12.82	29.45	7.93	3.43	12.42

WAZ: Weight-for-Age Z-score, LCL and UCL: Lower and Upper Confidence Interval Limits, (*n* = 145,002 children).

**Table 4:** Distribution of wasting prevalence and mean by state.

State	Mean WHZ			WHZ < 2%			WHZ −3 to −2%			WHZ < −3%		
	Estimation	95% LCL	95% UCL	Estimation	95% LCL	95% UCL	Estimation	95% LCL	95% UCL	Estimation	95% LCL	95% UCL
Al Gedaref	−0.50	−0.72	−0.28	10.98	5.33	16.62	8.38	2.12	14.64	2.35	0.01	5.04
Al-Gezira	−0.61	−0.77	−0.44	10.44	6.18	14.70	8.90	4.46	13.35	1.45	0.10	2.80
Blue Nile	−0.44	−0.61	0.27	7.02	3.25	10.79	6.01	2.10	9.92	0.92	0.01	2.12
Central Darfur	−0.82	−1.04	−0.60	16.46	9.03	23.89	12.65	4.64	20.66	3.65	0.16	7.14
East Darfur	−0.94	−1.12	−0.75	18.93	13.18	24.68	13.90	7.18	20.63	4.91	1.87	7.95
Kassala	−0.60	−0.76	−0.45	9.40	4.52	14.29	7.72	2.54	12.89	1.63	0.01	3.48
Khartoum	−0.81	−1.00	−0.61	14.61	9.98	19.25	11.92	6.79	17.04	2.56	0.75	4.37
North Darfur	−1.09	−1.39	−0.80	19.11	10.89	27.34	15.44	5.81	25.07	3.41	0.01	8.15
North Kordofan	−0.83	−0.97	−0.70	11.75	8.11	15.39	10.26	6.47	14.04	1.41	0.33	2.50
Northern	−0.88	−1.08	−0.68	16.46	11.19	21.72	12.82	7.12	18.53	3.47	0.92	6.02
Red Sea	−1.12	−1.40	−0.83	24.88	16.02	33.75	14.05	3.02	25.08	10.95	2.99	18.91
River Nile	−0.89	−1.08	−0.69	19.59	14.34	24.84	13.99	7.34	20.63	5.53	1.74	9.31
Sennar	−0.67	−0.82	−0.52	10.40	5.85	14.12	8.90	4.12	13.68	1.44	0.01	2.88
South Darfur	−0.92	−1.12	−0.71	15.03	8.50	21.56	12.38	5.41	19.36	2.59	0.01	5.33
South Kordofan	−0.51	−0.70	−0.33	7.83	3.26	12.39	6.78	2.29	11.27	0.94	0.01	2.20
West Darfur	−0.59	−0.79	−0.40	10.86	6.26	15.47	8.88	3.97	13.79	1.97	0.30	3.64
West Kordofan	−0.80	−0.95	−0.65	12.20	7.34	17.06	10.22	5.16	15.28	1.90	0.01	3.82
White Nile	−0.51	−0.66	−0.37	9.56	6.20	12.92	7.90	4.21	11.59	1.59	0.28	2.89
Sudan	−0.76	−0.96	−0.57	13.60	8.16	19.04	10.78	4.69	16.87	2.72	0.01	5.58

WHZ: Weight-for-Height Z-score, LCL and UCL: Lower and Upper Confidence Interval Limits, (*n* = 145,002 children).

**Table 5:** Distribution of MUAC wasting prevalence and mean by state.

State	Mean MUAC (mm)			MUAC < 125 mm GAM%			MAUC 115 mm–125 mm MAM%			MUAC < 115 mm SAM%		
	Estimation	95% LCL	95% UCL	Estimation	95% LCL	95% UCL	Estimation	95% LCL	95% UCL	Estimation	95% LCL	95% UCL
Al Gedaref	143.59	141.82	145.36	6.62	2.99	10.25	5.61	2.03	9.19	0.97	0.01	1.96
Al-Gezira	142.56	140.85	144.28	6.46	2.40	10.52	5.48	1.30	9.67	0.86	0.01	1.79
Blue Nile	142.02	140.36	143.69	8.63	5.21	12.04	7.08	3.40	10.75	1.51	0.24	2.78
Central Darfur	139.52	137.69	141.35	10.53	5.99	15.08	8.72	3.90	13.53	1.72	0.13	3.31
East Darfur	138.49	136.74	140.24	13.02	7.26	18.79	10.51	4.10	16.92	2.40	0.26	4.55
Kassala	139.54	138.05	141.02	12.45	8.46	16.44	9.79	5.03	14.55	2.73	0.88	4.58
Khartoum	146.16	144.24	148.08	5.24	2.04	8.44	4.33	1.04	7.62	0.83	0.01	1.76
North Darfur	136.79	134.94	138.64	15.86	9.59	22.13	12.43	5.60	19.26	3.22	0.75	5.69
North Kordofan	141.88	140.37	143.40	6.79	3.29	10.29	5.81	1.99	9.63	0.89	0.01	1.98
Northern	142.51	140.79	144.23	5.70	1.57	9.84	5.04	0.69	9.40	0.69	0.01	1.54
Red Sea	132.20	129.99	134.42	29.60	21.84	37.36	20.95	11.81	30.09	8.50	3.27	13.72
River Nile	140.95	138.97	142.93	10.65	4.63	16.66	8.38	2.21	14.55	1.98	0.01	4.55
Sennar	144.24	142.54	145.94	6.47	2.85	10.09	5.29	1.68	8.89	1.03	0.01	2.16
South Darfur	140.62	138.96	142.27	8.87	4.75	13.00	7.35	2.91	11.80	1.52	0.01	3.42
South Kordofan	140.39	138.26	142.52	9.78	5.08	14.49	7.88	2.81	12.95	1.86	0.09	3.63
West Darfur	140.06	138.63	141.49	9.02	4.90	13.14	7.58	3.66	11.50	1.34	0.19	2.50
West Kordofan	138.76	137.01	140.51	11.99	6.42	17.57	9.94	4.09	15.78	1.91	0.14	3.67
White Nile	141.99	140.40	143.58	7.97	2.71	13.22	6.34	1.05	11.64	1.19	0.01	2.75
Sudan	141.44	139.66	143.21	9.50	4.90	14.09	7.63	2.69	12.56	1.76	0.01	3.62

(*n* = 145,002 children), MUAC: Mid-upper-arm circumference, LCL and UCL: Lower and Upper Confidence Interval Limits, GAM: Global Acute Malnutrition, includes both MAM: Moderate Acute Malnutrition and SAM: Sever Acute Malnutrition.

## Data Availability

The data presented in this study are available on request from the authors.
